# Buccal mucosal membrane graft for correction of cicatricial lower eyelid retraction

**DOI:** 10.1186/s12886-022-02699-y

**Published:** 2022-12-01

**Authors:** Seyed Mohsen Rafizadeh, Seyed-Hashem Daryabari, Seyed Rahim Hassanpour

**Affiliations:** 1 Orbital and Oculoplastic Service, Farabi Eye Hospital, Tehran University of Medical Sciences, Tehran, Iran; 2 Chemical Injuries Research Center, Systems Biology and Poisonings Institutes, Baqiyatallah University of Medical Sciences, Tehran, Iran; 3 Eye Research Center, Farabi Eye Hospital, Tehran University of Medical Sciences, Tehran, Iran; 4 Department of Ophthalmology, Baqiyatallah University of Medical Sciences, Tehran, Iran

**Keywords:** Cicatricial lower eyelid retraction, Buccal membrane graft, Spacer graft

## Abstract

**Background:**

To evaluate the outcomes of a surgical technique using buccal mucosal membrane graft for correction of cicatricial lower eyelid retraction.

**Methods:**

Twelve patients with unilateral cicatricial lower eyelid retraction were enrolled in the study. All patients underwent a four-step surgical technique consisted of release of scars, midface lift, transfer of buccal mucosal membrane to posterior lamella as spacer graft, and canthal tightening. All patients were followed for at least 12 months.

**Results:**

Mean preoperative Margin-to-Reflex-Distance 2 (MRD2) was 7.73 ± 1.10 mm, compared to mean postoperative MRD2 of 5.04 ± 0.49 mm (P < 0.0001). The mean improvement in retraction was 2.69 mm. Postoperative scleral show was present in only one case and no major complications were observed.

**Conclusion:**

The four-step procedure (scar release, midface lift, buccal mucosal graft and canthal tightening) was an effective procedure to correct cicatricial lower eyelid retractions with acceptable outcomes and a low morbidity rate.

## Background

Lower eyelid retraction is defined as the inferior malposition of the lower eyelid margin with or without eyelid malrotation [1]. Cicatricial lower lid retraction can occur following various cosmetic or reconstructive procedures, such as lower blepharoplasty, orbital fracture repair, and eyelid reconstruction [2].

The primary mechanism is displacement of the lower lid margin away from the lower limbus due to septal scar, reduced orbicularis function, loss of volume and excessive skin excision. In some cases, these changes can progress and lead to lagophthalmos, ectropion, and poor cosmesis [3].

A thorough understanding of the complex lower lid anatomy is paramount for the treatment of lower lid malposition in eyelid surgery. The lower lid is composed of three distinct anatomical layers or lamellae [4] Cicatricial lower eyelid retraction primarily occurs as a result of contracture or relative shortage of the middle lamella, whereas cicatricial ectropion or entropion result from anterior or posterior lamellar scarring. Additionally, midface or sub orbicularis oculi fat (SOOF) descent in conjunction with cicatricial lower eyelid changes and large eye morphology cause severe lower eyelid malposition [1].

Lower lid retraction usually warrants surgical correction. A variety of surgical techniques have been used, ranging from relatively simple procedures, such as a full-thickness skin graft or myocutaneous switch flaps, to more complicated operations, such as middle and posterior lamella lengthening, midface lifting, and spacer grafts [5] Spacer materials may include hard palate mucosa, buccal mucous membrane, dermis-fat grafts, tarsoconjunctival autografts, auricular cartilage, donor sclera, acellular dermis, and alloplastic materials [6].

The optimal surgical method for correction of cicatricial lower lid retraction still remains a matter of controversy. In this study, we describe our experience with a four-step surgical technique abbreviated as “RETRACT”: Release of scars, Elevation of midface, TRAnsfer of buccal mucous membrane to posterior lamella as the spacer graft, and Canthal Tightening.

## Methods

This prospective interventional study was performed in accordance with the tenets of the Declaration of Helsinki and was approved by Baqiyatallah University of Medical Sciences Research Ethics. Consecutive patients referred to the oculoplastic clinic of a tertiary ophthalmology center with unilateral cicatricial lower eyelid retraction between 2018 and 2020 were enrolled. Patients were included only if lower eyelid retraction was cicatricial and there were no other etiologies of retraction such as Grave’s orbitopathy. Written informed consent was obtained from all participants included in the study.

All patients underwent complete ophthalmologic evaluation, including determination of visual acuity, intraocular pressure and slit-lamp examinations. Patient characteristics including age, sex, laterality, proposed cause, degree of scleral show and Margin-to-Reflex Distance 2 (MRD2) were recorded (Table 1).

**Table 1:** Preoperative and Postoperative Data of All Patients

Patient No.	Age	Sex	Side	Cause of Lower Eyelid Retraction	Degree of Scleral Show*	Margin-to-Reflex Distance 2 (MRD2)		
						Preoperative	Postoperative	Change
1	46	F	L	Multiple times Cosmetic Injections (Fat & Filler)	Moderate	7.5	5	-2.5
2	20	M	L	Surgery for Orbital Floor Fracture	Mild	6	4	-2
3	56	M	L	Trauma & Lower lid & Cheek lacerations	Severe	10	6	-4
4	37	M	L	Childhood Trauma	Severe	8	5	-3
5	33	F	R	Lower Lid Transcutaneous Blepharoplasty	Severe	8.5	5	-3.5
6	58	M	R	Surgery for Orbital Floor Fracture	Severe	8.5	5.5	-3
7	25	M	L	Surgery for Orbital Floor Fracture	Mild	6.5	5	-1.5
8	42	F	L	Childhood Trauma	Moderate	7.5	5	-2.5
9	38	M	L	Surgery for Maxillofacial Fracture	Severe	8	5	-3
10	38	F	R	Surgery for Maxillofacial Fracture	Moderate	7	5	-2
11	37	F	R	Surgery for Orbital Floor Fracture	Mild	6.5	4.5	-2
12	31	F	R	Old Inflammation and two previously failed procedures for correction of retraction	Severe	8.5	5.5	-3

*F* Female, *M* Male, *L* Left, *R* Right

*Degree of Scleral show: Mild, <1 mm; Moderate, 1 to 2 mm; Severe, >2 mm

Margin-to-Reflex Distance 2 (MRD2) was defined as the distance of the pupillary light reflex from the superior edge of the inferior eyelid and was measured in millimeters. Inferior scleral show was categorized as mild (up to 1 mm), moderate (1 to 2 mm) or severe (more than 2 mm). Preoperative and 12-month postoperative digital photographs were obtained in primary gaze.

All patients underwent the RETRACT procedure by a single oculoplastic surgeon under general anesthesia and were followed for at least 12 months postoperatively.

### Surgical technique: the RETRACT surgery

#### 1^st^ step: Release of scars

First, lateral canthotomy and inferior cantholysis were performed and the lower limb of lateral canthal tendon was cut. A 4 − 0 silk suture was passed through the margin of the eyelid to provide upward vertical traction on the lower eyelid, allowing identification of the scar. Then, the lower eyelid was everted and a full-thickness horizontal incision was fashioned through the posterior surface of the eyelid, approximately 1 mm below the lower end of tarsus. Lower lid retractors (capsulopalpebral fascia) were disinserted from the tarsus and another 4/0 silk suture was passed through the inferior edge of conjunctiva and retractors and pulled superiorly for better exposure. Dissection was performed to completely release any scar tissue tethering the lid to the orbital septum and inferior orbital rim, while upward traction was applied to the lower eyelid to assess the degree and site of residual scarring, which was subsequently released.

#### 2^nd^ Step: Elevation of midface

**Fig. 1:**
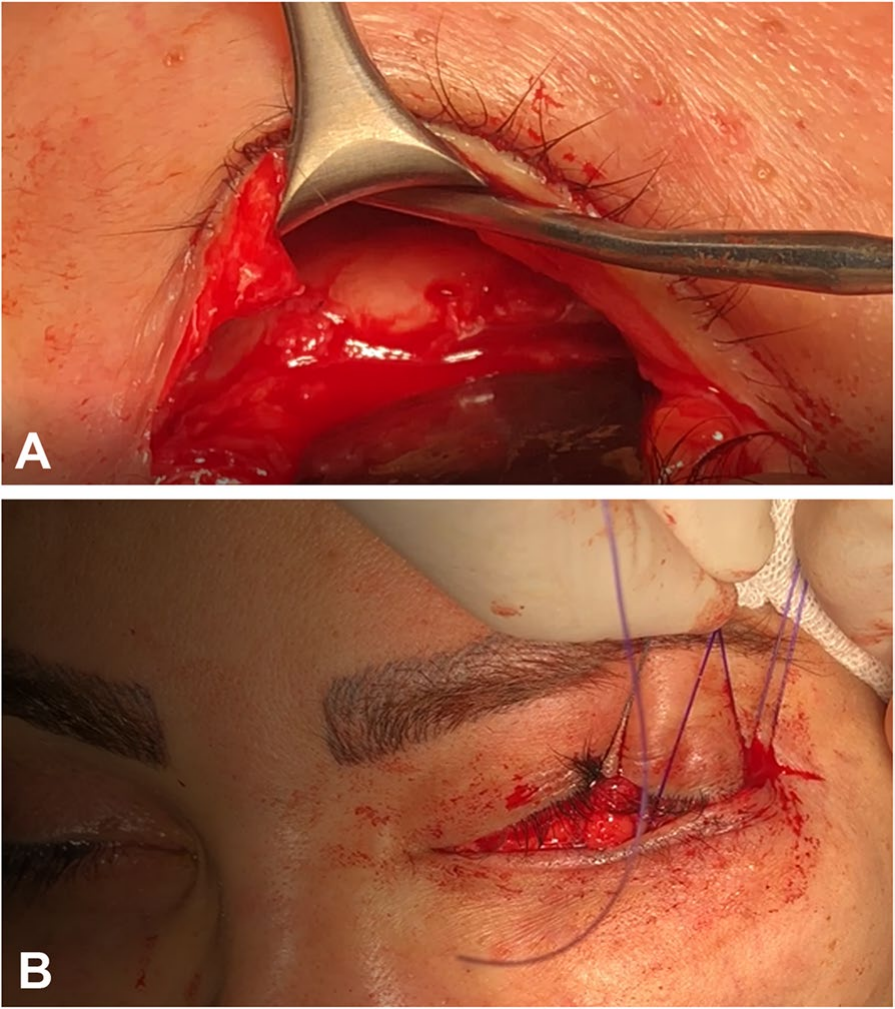
**A **Intraoperative photograph showing subperiosteal dissection extended about 2–3 cm below the inferior orbital rim to prepare tissues for midface elevation. **B** Grasping the superficial musculoaponeurotic system (SMAS) and suborbicularis oculi fat pad (SOOF) with a 3 − 0 PDS suture (Ethicon Inc, NJ, USA) for midface elevation to its proper position for fixation to the lateral orbital rim periosteum

In moderate and severe cases of retraction, the periosteum of inferior orbital rim was cut and elevated, followed by subperiosteal dissection for about 2 cm below the inferior orbital rim (Fig. 1A). In mild cases and those who had previous repair of inferior orbital rim fracture using titanium plate, preperiosteal dissection was performed. Once the midface had been freed and was easily mobile, the superficial musculoaponeurotic system (SMAS) and/or suborbicularis oculi fat pad (SOOF) was taken with a 3 − 0 PDS suture (Ethicon Inc, NJ, USA) and fixed to the lateral orbital rim periosteum tightly (Fig. 1B). In moderate and severe cases, the elevated periosteum was also taken through the suturing and fixation.

#### 3^rd^ step: TRAnsfer of buccal mucous membrane to posterior lamella as the spacer graft

**Fig. 2:**
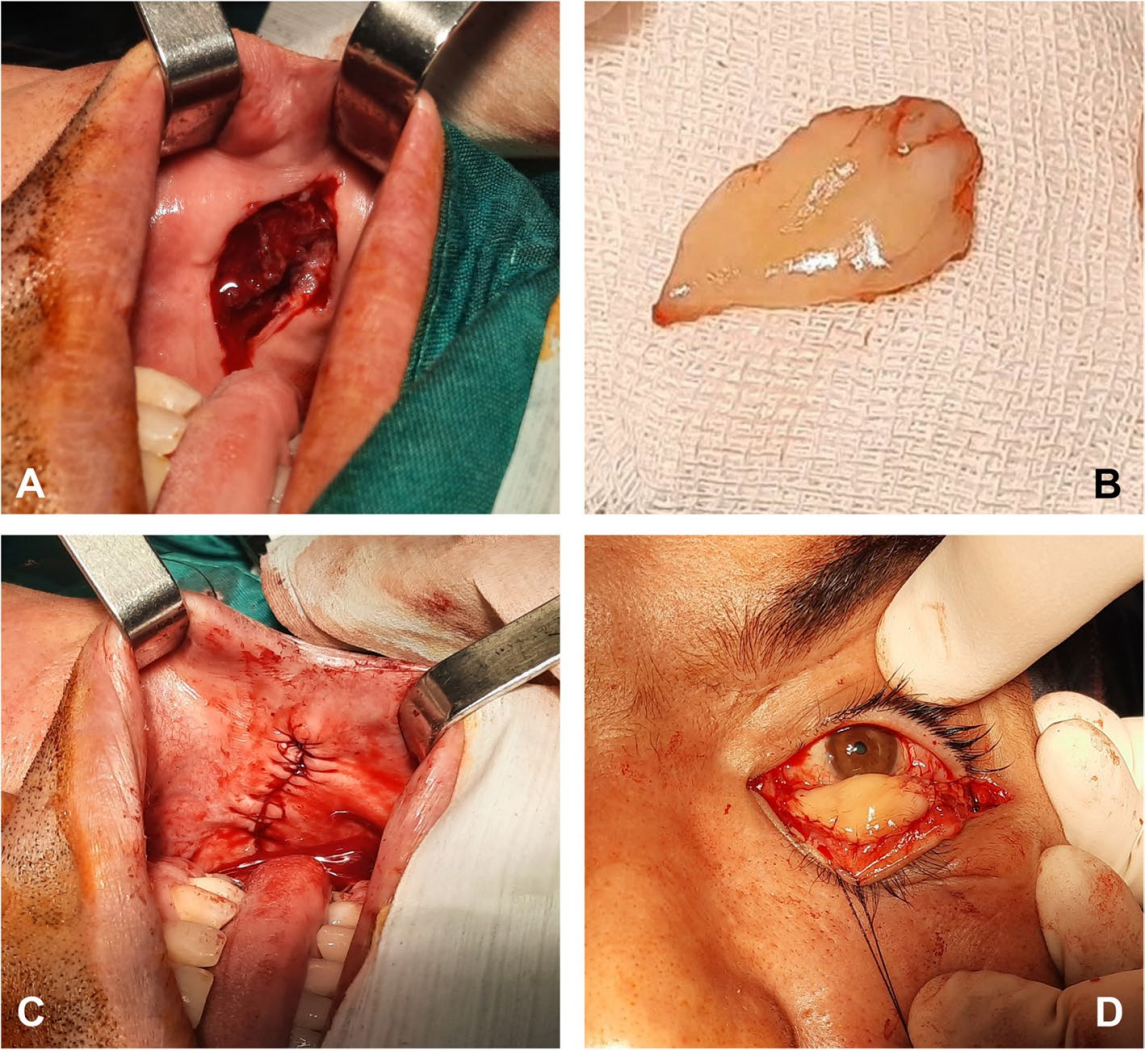
A, Intraoperative photograph showing buccal mucosal membrane donor site after harvesting graft. B, Gross appearance of buccal mucosal membrane graft (BMMG) which is initially harvested twice the size of the conjunctival defect to compensate for postoperative shrinkage. C, Photograph of the repaired donor site with a 5 − 0 Vicryl suture (Steinerberg 8, Belgium). D, BMMG fixed to the posterior lamella between the lower lid retractors and tarsal plate using 6 − 0 Vicryl suture (Steinerberg 8, Belgium)

After marking buccal mucous membrane in one of the lateral sides of mouth twice the size of the defect of posterior lamella, the marked area was incised using a number 15 blade and dissected with a Stevens scissors (Fig. 2A, B). The defect at the donor site was repaired by a 5 − 0 long needle Vicryl suture (Steinerberg 8, Belgium) (Fig. 2C). The graft was then transferred to the posterior surface of the eyelid with the mucosal side facing the globe. The graft was sutured to the posterior lamella using 6 − 0 Vicryl sutures (Steinerberg 8, Belgium) (Fig. 2D). Two anchoring sutures were used by passing a 4 − 0 Polypropylene suture (Steinerberg 8, Belgium) through the bed of the graft to lower lid skin and were fixed using bolsters.

#### 4^th^ step: Canthal Tightening

In the final step, lateral canthopexy was performed using a double armed 4 − 0 Polypropylene suture (Steinerberg 8, Belgium) through the lateral end of tarsus by fixing it to the inner aspect of the lateral orbital rim periosteum, without fashioning a tarsal strip. Then, the skin incision of lateral canthus area was repaired with a 6 − 0 Nylon suture. Nylon skin sutures as well as the anchoring sutures were removed after 2–3 weeks.

At the end of procedure, the Frost suture passing through the lower lid margin was taped on the forehead to provide adequate upward traction, lasting 4–5 days. The postop medications included topical 0.5% chloramphenicol and 0.1% betamethasone eye drops four times a day for 2–3 weeks and 500 mg oral cephalexin every 6 h for one week.

All patients were followed at 1, 3, 6 and 12 months after the surgery and the main final outcome measure (MRD-2) was recorded at the 12-month postop visit. Other information like lagophthalmos, dry eye symptoms, subjective patient satisfaction and complications related to the donor site or the eye were also gathered.

Statistical analysis was performed using SPSS V 23.0 (IBM, Armonk, NY). For assessment of significance of difference between pre- and post-op MRD-2, paired t-test was used and *P* value < 0.05 was considered to indicate statistical significance.

## Results

Twelve patients (6 males and 6 females) were included in the study. The mean patient age was 38.4 ± 11.1 years (range, 20–58 years). Etiology of the lower lid retraction was: surgery for orbital floor fracture in 4 cases (33.3%), orbital trauma in 3 (25%), maxillofacial fracture surgery in 2 (16.6%), and one case with either of the following: cosmetic injections (8.3%), lower lid transcutaneous blepharoplasty (8.3%) and old inflammation (8.3%).

Degree of preoperative inferior scleral show was mild, moderate and severe in 3 (25%), 3 (25%), and 6 (50%) patients, respectively.

Follow-up ranged from 12 to 18 months. All patients had significant improvement in eyelid retraction, lagophthalmos, and self-assessed dry eye symptoms. Subjective patient satisfaction was high in all cases.

**Fig. 3:**
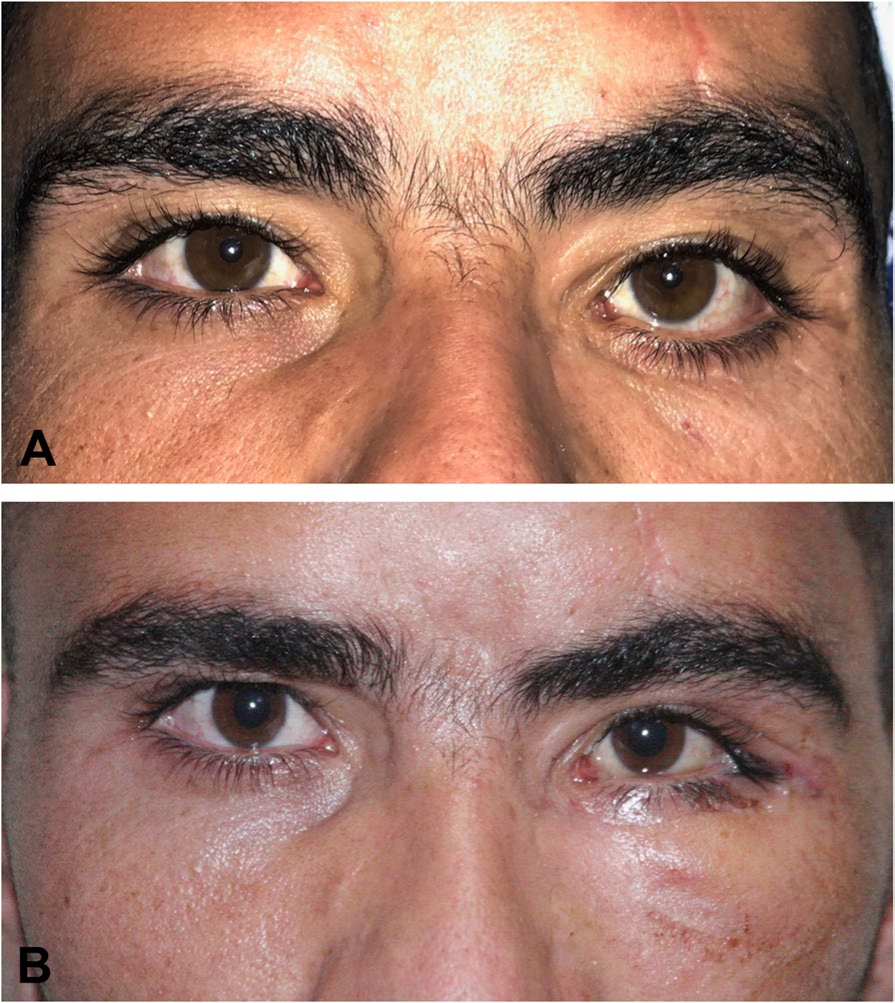
**A **Preoperative photograph of patient No.7 showing mild cicatricial left lower lid retraction due to previous surgery for orbital floor fracture. **B** 3-month postoperative photograph of the same patient showing return of lower lid to normal position

**Fig. 4:**
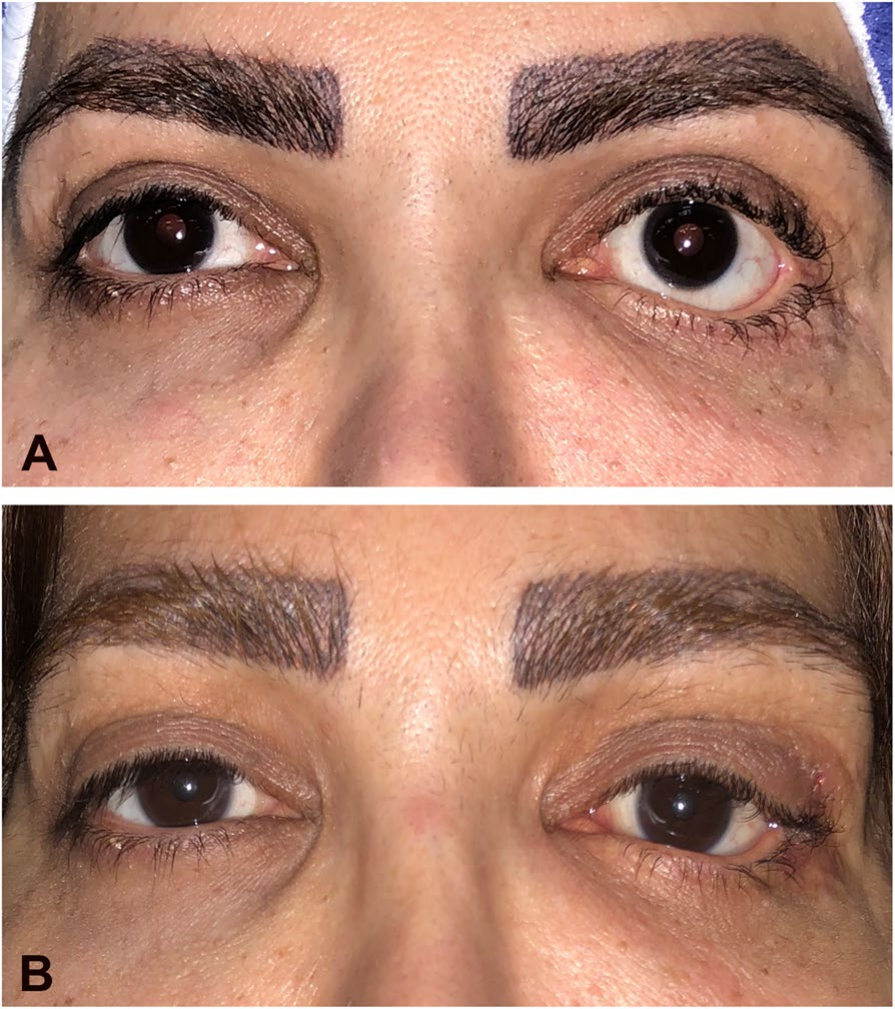
**A **Preoperative photograph of patient No.1 showing moderate cicatricial left lower lid retraction due to multiple cosmetic fat and filler injections. **B** 6-month postoperative photograph of the same patient showing notable improvement in scleral show

**Fig. 5:**
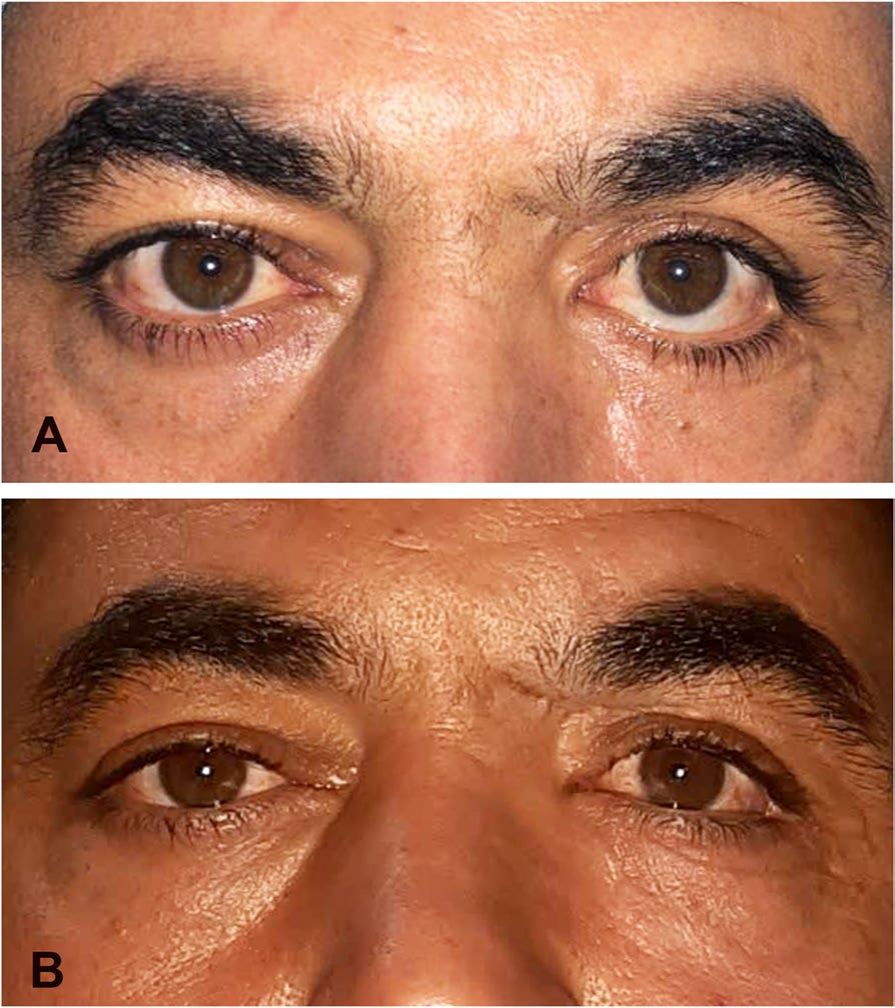
**A** Preoperative photograph of patient No.9 showing severe cicatricial left lower lid retraction after surgery for maxillofacial fracture. **B** 12-month postoperative photograph of the same patient showing marked improvement in lower eyelid retraction and scleral show

The mean preoperative MRD2 was 7.73 ± 1.10 mm (range, 6–10 mm) and the mean postoperative MRD2 was 5.04 ± 0.49 mm (range, 4–6 mm; *P* < 0.0001). The mean improvement in retraction was 2.69 mm (range, 1.5-4 mm) in the whole cohort, 1.83 mm in mild, 2.33 mm in moderate and 3.25 mm in severe cases (Figs. 3, 4 and 5).

There was a significant relationship between preoperative scleral show and retraction improvement after surgery, with greater improvement in severe cases compared to moderate and mild cases.

There was no postoperative scleral show nor MRD2 greater than 5.5 mm except in one patient who had extremely severe retraction and scleral show with 10 mm preoperative MRD2 and extensive notable adhesions and scars during procedure. However, this patient was also highly satisfied with the outcome of the operation.

There were no major complications following this procedure and none of the patients needed a second operation. There were some minor complications including ocular redness and irritation in 3, slight buccal pain in 2, and ocular watery discharge and tearing in 1 patient. All lasted for less than 2 weeks and completely resolved with usual conservative treatments.

## Discussion

Correction of lower eyelid retraction is usually challenging because it involves different pathologies and addressing all of them is necessary to achieve acceptable outcome. Cicatricial retraction is even more challenging compared to other causes such as Grave’s orbitopathy, because the normal anatomy has been violated and scars and adhesions further complicate the corrective measures.

Deep understanding of eyelid and periorbital anatomy and etiologies of lower lid retraction is necessary to make the proper choice of surgical methods that resolve the underlying pathologies. As mentioned before, lower lid is being composed of three distinct anatomical layers: the anterior lamella consists of the thinnest skin in the body with no underlying subcutaneous fat and also orbicularis oculi muscle, the middle lamellae include orbital septum which is the most important layer in cicatricial retraction, and the posterior lamellae composed of the tarsus superiorly and the lower eyelid retractors inferiorly and the conjunctiva [4].

Understanding the anatomic relationship of the lower eyelid–midface unit is important in recognizing the role midface descent can play in lower lid malpositioning. As the midface ages, there is bony remodeling, descent of the malar fat pad secondary to gravity, as well as fat atrophy, which result in lower eyelid descent and malposition due to loss of support [7].

Involvement of any of the three layers of the eyelid can lead to lower lid retraction especially in conjunction with horizontal lid laxity, canthal dystopia, or malar ptosis caused by aging that reduces the intrinsic lower lid support. However, involvement of middle lamella (the orbital septum) plays the most important role in severity of retraction and is almost always present in moderate and severe cases [4].

Middle lamella scarring refers to fibrosis between the orbital septum and the capsulopalpebral fascia or lower eyelid retractors. Disruption of the orbital septum iatrogenically or traumatically results in contracture. Contracture and disruption of the septum can lead to fusion of these layers. Also, inflammation of the orbital fat pads can cause fusion. This scar band can lead to a more hollowed eye appearance by posterior displacement of the orbital fat pads. This displacement will ultimately retract the lower lid inferiorly oftentimes resulting in scleral show. Releasing the capsulopalpebral fascia from the scarred orbital septum is the goal for treatment of middle lamella scarring [7, 8].

Different procedures have been proposed to correct lower eyelid retraction, including lateral canthal surgery (canthoplasty or canthopexy), transverse tarsotomy and tarsoconjunctival flap, autogenous spacers, retractor release, cheek suspension techniques and usage of fillers [2, 4–6, 9–19]. In mild cases, typically a single underlying etiology exists that usually is sufficiently resolved by a simple procedurec. However, all the 3 patients with mild scleral show in our study had previous surgery for inferior orbital floor fracture and many scars and adhesions that prevent the inferior eyelid to return to its proper position with a simple procedure. On the other hand, we used the examinations that Patipa [20] described for evaluation of lower eyelid retraction and all the 3 patients needed 3 or 4 fingers to reposition the lower eyelid to its proper position indicating that simple surgery would not be successful. Otherwise, in moderate and severe cases and particularly in cicatricial retractions, numerous pathologies are involved and a combination of several methods should be used to achieve optimal outcome.

Our suggested combined procedure addresses four major factors involved in cicatricial lower eyelid retraction, namely scars and adhesions, midface descent, middle and posterior lamellar shortening and lateral canthal tendon laxity.

Midface elevation is commonly used to correct lower lid retraction and has even been reported to be employed as the only procedure required in some cases [21]. It is particularly helpful when the problem affects the anterior lamella, as a proper midface lift recruits more skin and eliminates the need for an extra skin graft. Our approach to the midface was through the transconjunctival incision between lower edge of tarsus and retractors which obviated the need for a separate incision.

Surgical correction of the lower lid retraction frequently requires a spacer graft to keep the retractors recessed and support the tarsus in an upward position. The role of spacer grafts is more accentuated in cases with lower eyelid middle and posterior lamella scar or shortage in providing additional augmentation by lengthening the lower lid retractors and supporting the lower eyelid following release of the cicatrix [1].

Currently, a large number of grafts are used as spacers in middle and posterior lamellae, including homologous tissues (e.g., hard palate, ear cartilage, nasal septal cartilage, tarsus, periosteum, temporalis fascia, fascia lata, and sclera), and alloplastic implants (e.g., human cadaveric acellular dermis (AlloDerm; LifeCell Corp), decellularized porcine-derived membrane (tarSys; IOP Inc), high density polyethylene (Medpor), Mersilene mesh (Ethicon), and polytetrafluoroethylene (PTFE)) [2–5, 7, 9, 10, 13, 14, 16, 18, 19, 22–24].

The ideal spacer graft should be biocompatible and easily accessible with a low rate of contracture and some degree of stiffness to provide support. Additionally, it should promote tissue integration with minimal inflammation and allow mucosalization on the conjunctival side. Ideally, it should have thickness, rigidity and contour characteristics that approximate the lower lid tarsus, and a mucosal surface so as not to irritate the corneal surface and have low risk of rejection and absorption [3, 25]. We found BMMG to be a suitable and accessible material without the disadvantages and limitations of other spacers.

BMMG, has several advantages over other spacer graft materials, including readily accessible mucosa, absence of keratinizing epidermis or dermal appendages, and an easily vascularized surface from the surrounding conjunctiva to promote graft survival. Being an autologous tissue, it imposes less cost to the patient compared to alloplastic materials and lacks the risk of transmitting infections. Nevertheless, some surgeons still prefer alloplastic materials because of lack of the need for harvesting procedures that prolong the operating time and bear the potential for donor-site morbidity.

In our study, donor site morbidity was minimal. All patients began oral feeding after 1 or 2 days after surgery mostly with fluids, returned to regular diet in less than 1 week and recovered without scarring or deformity within 2–3 weeks. Our results were more similar to Kim et al. [26], who published on their experience with buccal mucosal graft for anophthalmic socket reconstruction of 44 patients. Their subjects had only minor and tolerable morbidity of the donor site and recovered without deformities or scars. Kumar et al. [27] reported that patients who underwent buccal mucosa harvesting had problems with oral feeding and were not able to resume their regular activities until 1 month postoperatively. On the other hand, Neuschl et al. [28] concluded that short- to medium-term donor site morbidity is tolerable and long-term donor site morbidity is rare.

Postoperative graft shrinkage and contracture are possible in BMMG, but the rate is lower than other materials. Mean graft contraction rates have been 57% for acellular dermis and 16% for hard palate mucosal graft in lower eyelid surgery [29]. Graft contracture has been reported in only 4.5% of socket reconstructions using BMMG, less than any other spacer grafts [26]. In our study no graft contracture was observed during follow up. As postoperative contraction of the mucosal surface usually develops within 6 months after implantation, we believe further late-onset graft contracture or shrinkage in our cases would be highly unlikely.

Previous studies on surgical treatment of lower eyelid retraction have showed varying outcomes.

Oestreicher et al. [18] compared the outcome of three different posterior lamellar grafts (including hard palate mucosa, free tarsoconjunctival, and free scleral grafts) in a retrospective study of 659 eyelids of 400 patients with four retraction etiologies. They reported a mean reduction of 1.3 mm in scleral show. Korn et al. [23] achieved a mean improvement of 2.03 mm in MRD2 in 16 eyes of 11 patients who underwent midfacial lifting and dermis fat as a spacer. The patients had different types and etiologies of retraction. In 24 retracted eyelids of 17 patients, Patel et al. [4] described an average of 2.5 mm of scleral show improvement using a surgical technique involving hard palate graft placement, canthopexy, and midface suspension. In another study, Patel et al. [11] described 17 patients with post blepharoplasty lower eyelid retraction who had a mean improvement in scleral show of at least 1.8 mm after hard palate graft placement and lateral tightening. Wearne et al. [15] reported 102 eyelids of 68 patients treated with hard palate graft who had a mean reduction of 2.3 mm of scleral show.

In comparison, the average 2.66 mm reduction in MRD2 of our patients, is equal and slightly higher than the previous studies discussed. Also, we had residual scleral show in only one patient (8.3%), a rate that is lower than some of the studies.

These results support the efficacy of our surgical technique and spacer graft. However, the small sample size of our patients limited to only cicatricial retraction cases, can affect our findings. There are other important limitations to our study like lack of a comparison group or blinding of the researchers and the patients.

## Conclusion

In conclusion, the RETRACT surgery, which is combination of scar release, elevation of midface, transfer of buccal mucosal membrane graft to the posterior lamella as a spacer, and lateral canthal tightening, is an effective procedure to correct cicatricial lower eyelid retractions with acceptable outcomes and a low morbidity rate.

## Data Availability

All data generated or analyzed during the current study are included in this published article except for face photos of some patients that are not publicly available because it was not necessary for the article and we didn’t obtain consent from them to publish their photos publicly. But they would be available from the corresponding author on reasonable request after obtaining relevant consent.

## Abbreviations

SOOF: Sub Orbicularis Oculi Fat
RETRACT surgical technique: Release of scars, Elevation of midface, TRAnsfer of buccal mucous membrane to posterior lamella as the spacer graft, and Canthal Tightening
MRD-2: Margin-to-Reflex Distance 2
SMAS: Superficial Musculoaponeurotic System
PTFE: Polytetrafluoroethylene
BMMG: Buccal Mucosal Membrane Graft
